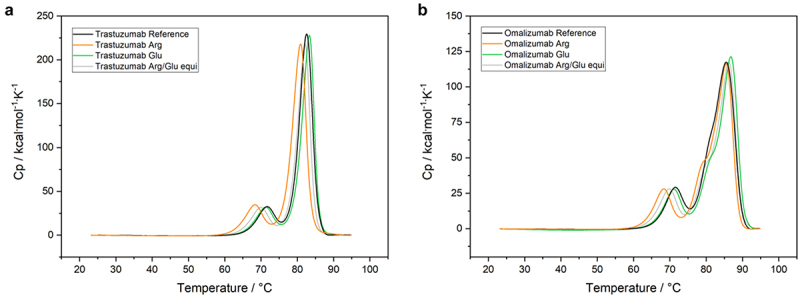# Correction

**DOI:** 10.1080/19420862.2024.2436775

**Published:** 2024-12-04

**Article title**: Residue-resolved insights into the stabilization of therapeutic proteins by excipients: A case study of two monoclonal antibodies with arginine and glutamate

**Authors**: Prass, T. M., Garidel, P., Schäfer, L. V. & Blech, M.

**Journal**: *mAbs*

**DOI**: https://doi.org/10.1080/19420862.2024.2427771

The author has been notified that the x-axis annotations in Figures 1 and 2 do not appear to be entirely accurate, possibly due to the use of an older version of the figures. The author has requested that the current versions of Figures 1 and 2 be replaced with the updated versions provided below, as they more accurately reflect the original intentions.

**Figure 1:**
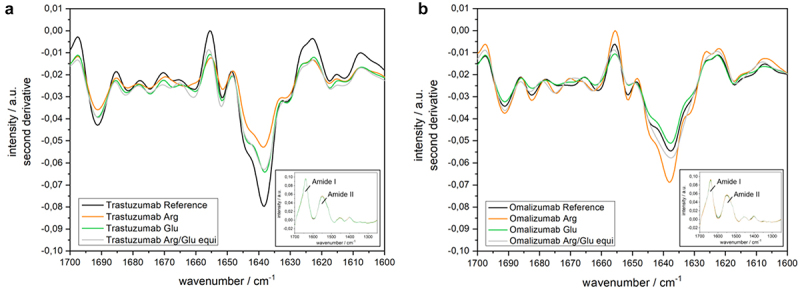

**Figure 2:**